# Image-Guided Hypofractionated Radiotherapy in Low-Risk Prostate Cancer Patients

**DOI:** 10.1155/2014/465175

**Published:** 2014-04-23

**Authors:** Maurizio Valeriani, Alessia Carnevale, Linda Agolli, Paolo Bonome, Adelaide Montalto, Luca Nicosia, Mattia F. Osti, Vitaliana De Sanctis, Giuseppe Minniti, Riccardo Maurizi Enrici

**Affiliations:** Department of Radiation Oncology, “La Sapienza” University, Sant'Andrea Hospital of Rome, Via di Grottarossa 1035-1039, 00189 Rome, Italy

## Abstract

*Aim*. To evaluate efficacy and toxicity of image-guided hypofractionated radiotherapy (HFRT) in the treatment of low-risk prostate cancer. Outcomes and toxicities of this series of patients were compared to another group of 32 low-risk patients treated with conventional fractionation (CFRT). *Methods*. Fifty-nine patients with low-risk prostate cancer were analysed. Total dose for the prostate and proximal seminal vesicles was 60 Gy delivered in 20 fractions. *Results*. The median follow-up was 30 months. The actuarial 4-year overall survival, biochemical free survival, and disease specific survival were 100%, 97.4%, and 97.4%, respectively. Acute grade 1-2 gastrointestinal (GI) and genitourinary (GU) toxicity rates were 11.9% and 40.7%, respectively. Grade 1 GI and GU late toxicity rates were 8.5% and 13.6%, respectively. No grade ≥2 late toxicities were recorded. Acute grade 2-3 GU toxicity resulted significantly lower (*P* = 0.04) in HFRT group compared to the CFRT group. The cumulative 4-year incidence of grade 1-2 GU toxicity was significantly higher (*P* < 0.001) for HFRT patients. *Conclusions*. Our study demonstrated that hypofractionated regimen provided excellent biochemical control in favorable risk prostate cancer patients. The incidence of GI and GU toxicity was low. However, HFRT presented higher cumulative incidence of low-grade late GU toxicity than CFRT.

## 1. Introduction

Hypofractionated radiation therapy (HFRT) has been suggested as an attractive strategy of treatment to improve results in localized prostate cancer. In contrast to other tumors, prostate cancer seems to have a low *α*/*β* ratio [1]. Thus, a therapeutic gain could be obtained by irradiating patients using schedules with larger dose per fraction and lower number of fractions. In addition to a possible radiobiological benefit, hypofractionated RT allows a shorter overall treatment time and reduction of treatment costs. Randomized trials have shown a better biochemical control when higher total doses of conventionally fractionated irradiation (CFRT) are delivered to the prostate [2].

In our study, we assessed the acute and late toxicity and the biochemical control in patients with low-risk prostate cancer receiving external beam radiation therapy (EBRT) using a hypofractionated schedule. Furthermore, toxicity rates were compared between this series and another group of patients who underwent standard fractionation regimen.

## 2. Materials and Methods

### 2.1. Patients' Characteristics

Between January 2007 and January 2013, 59 patients with biopsy proven, low-risk prostate cancer were treated with HFRT therapy associated with IGRT. Median age at diagnosis was 72 years (range 48–82 years). All patients presented cT1/2a N0 M0 clinical stage, a Gleason score of 6, and a pretreatment prostate-specific antigen (PSA) serum level <10 ng/mL.

Pretreatment evaluation included complete physical examination, PSA level, complete blood counts and standard biochemistry tests, bone scan, total body computed tomography (CT) with contrast medium, and prostate magnetic resonance image (MRI) with diffusion and perfusion sequences.

Median of PSA value at diagnosis was 5.94 ng/mL (range 2.6–9.4 ng/mL). Outcomes and toxicity profile of patients receiving HFRT were compared with a group of 32 low-risk patients treated with CFRT and image-guided radiotherapy (IGRT) that refused hypofractionated treatment. Median of PSA value at diagnosis for CFRT patients was 6.4 ng/mL (range 3.1–9.6 ng/mL). All patients provided written informed consent. Patient characteristics are summarized in Table 1.

### 2.2. Treatment

All patients underwent a pretreatment CT planning (2.5 mm slice thickness) in the supine position with feet rests for the implementation of treatment planning. The preparation for CT scan encompassed the administration to a mini enema for rectal emptying and then patients were invited to next urination. In addition, they were requested to drink 500 mL of water half an hour before the start of the procedure to fulfill the bladder. Planning CT images were fused with MRI images (diffusion ADC map, perfusion series, and axial high resolution T2-w) using point-to-point matching to help clinical target volume (CTV) delineation.

The CTV included the prostate and the first centimeter of the seminal vesicles. Planning target volume (PTV) was generated adding a 5 mm margin in all directions. The whole rectum from the anus to the sigmoid flexure, bladder, femoral heads, and penile bulb were delineated as organs at risk.

A 3D conformal plan on the Eclipse planning system (Varian, Palo Alto, CA) was performed using 5 coplanar fields. Treatment was delivered by a linear accelerator using 15 MV photons. Thus, the PTV received 60 Gy in 20 fractions (3 Gy per fraction) in the hypofractionated group and 76 Gy in 38 fractions (2 Gy per fraction) in the conventional group, five weekly times. According to the Linear Quadratic Model, the hypofractionated regimen is biologically equivalent to 77.1 Gy in 2 Gy fractions assuming an *α*/*β* ratio of 1.5 Gy. This regimen is also equivalent to 72 Gy in 2 Gy fractions assuming an *α*/*β* ratio of 3 Gy for late responding tissue. Dose-volume constraints were as follows: V50 < 35% and V58 < 25% for the rectum; V43 < 50% for the bladder.

Prior to each treatment, patients underwent a Kilo-voltage cone-beam CT that was compared with the planning CT to verify the correct position. The patients' position was adjusted with an initial automatic bone alignment, followed by a soft tissue alignment using the prostate-rectum interface.

From the start of radiation therapy, all patients were advised to follow a low-fibre and low-fat diet and to assume a cranberry based integrator and lactic ferment once daily.

### 2.3. Toxicity and Follow-Up

Follow-up was performed every 3 months for the first year and every 6 months afterwards. Toxicities were assessed at each follow-up according to the Radiation Therapy Oncology Group (RTOG) scale for acute and late adverse effects [3]. Late toxicities were considerate after 90 days from the RT completion.

### 2.4. Statistical Analysis

The biochemical failure was defined as the PSA nadir + 2 ng/mL according to the Phoenix criteria [4]. Overall survival (OS), disease specific survival (DSS), and biochemical free survival (bNED) were calculated to the event using the Kaplan-Meier method. Difference in the cumulative incidence of ≥grade 2 late toxicities between the two groups (presence of toxicity at any time of follow-up was considered as event) was evaluated with log-rank test. In the subgroups analysis, acute toxicities between HFRT and CFRT groups were compared using the chi-square test. Statistical analysis was performed using SPSS statistical software package version 13.0 (SPSS, Inc., Chicago, IL). A *P* value lower than 0.05 was considered as statistically significant.

## 3. Results

### 3.1. Survivals and Relapse

The median follow-up for patients treated with HFRT was 30 months (range 12–76 months), whereas for patients treated with CFRT it was 52 months (range 7–79 months). The overall median follow-up was 40 months (range 7–76 months).

The median PSA value at last follow-up was 0.51 ng/mL (range 0.04–3.1 ng/mL) in the HFRT group. The median PSA value at last follow-up was 0.42 ng/mL (range 0.04–1.5 ng/mL) in the CFRT group.

The actuarial 4-year OS was 100% and 93.8% (*P* = 0.053) for the HFRT and CFRT groups, respectively. Two deaths occurred in the CFRT group at the time of the statistical analysis. The patients died after 7 and 10 months from RT completion, respectively, for cardiopulmonary disorders without any evidence of disease. The actuarial 4-year bNED and DSS were 97.4% for HFRT group. The actuarial 4-year bNED and DSS were 100% for the CFRT group. There was no statistical difference (*P* = 0.374) regarding survivals between the two groups. One patient developed biochemical failure with evidence of external iliac lymph nodes involvement confirmed by Choline TC-PET in the HFRT group.

### 3.2. Acute Toxicities

Toxicities occurred as follows during treatment: grade 1 and grade 2 gastrointestinal (GI) toxicity in 14/91 (15.4%) and 2/91 patients (2.2%), respectively; grades 1, 2, and 3 genitourinary (GU) toxicity in 38/91 (41.8%), 6/91 (6.6%), and 1/91 patients (1.1%), respectively.

At 3 months after radiation treatment grade 1 GI toxicity was observed in 5/91 patients (5.5%), and grade 1 GU toxicity was observed in 9/91 patients (9.9%). No grade ≥2 GU or GI toxicities were recorded.

No statistically significant difference was calculated between the two groups treated with CFRT and HFRT at 3 months from the end of therapy. During treatment grade 2-3 GU toxicity resulted statistically higher (*P* = 0.04) in the group treated with CFRT. Toxicity rates are summarized in Table 2.

### 3.3. Late Toxicities

At 6 months from the end of therapy, 2 patients (3.4%) treated with HFRT and 4 (12.5%) treated with CFRT presented grade 1 GI toxicity. Grade 2 GI toxicity was observed in 1 patient (3.1%) that received CFRT treatment. Seven patients (11.9%) treated with HFRT and 3 (9.4%) treated with CFRT presented grade 1 GU toxicity. No grade ≥2 toxicities were recorded.

At the last follow-up, grade 1 GI and GU toxicities were observed in 5 (8.5%) and 8 (13.6%) patients treated with HFRT, respectively. Patients treated with CFRT experienced grade 1 GI and GU toxicity in 12.5% and 3.1% (1 patient), respectively. Only one patient (1.1%) developed grade 2 GI toxicity (CFRT group). No grade ≥3 toxicities were recorded.

**Figure 1:**
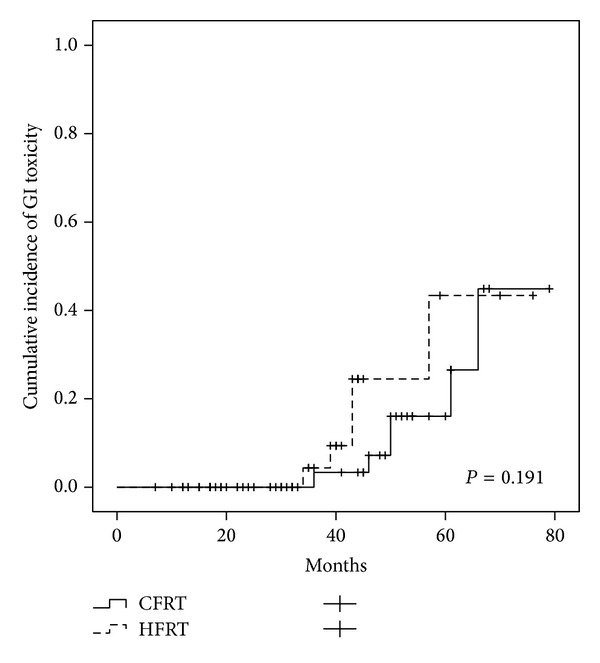
Comparison of grade 1-2 GI late toxicity in the CFRT and HFRT group.

**Figure 2:**
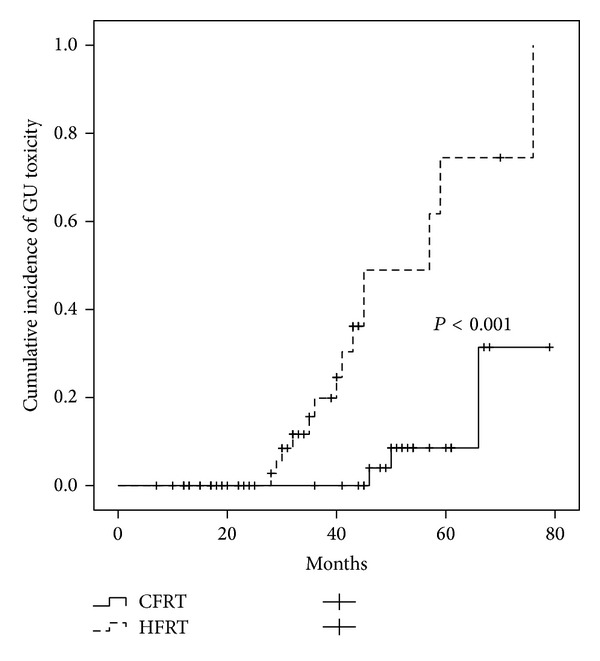
Comparison of grade 1-2 GU late toxicity in the CFRT and HFRT group.

The cumulative incidence of grade 1-2 GI toxicities at 4 years was 13.6%, for CFRT group was 7.2%, and for HFRT group was 24.5% (*P* = 0.191). The cumulative incidence of grade 1-2 GU toxicities at 4 years was 11.5%, for CFRT group was 4%, and for HFRT group was 49% (*P* < 0.001) (Figures 1 and 2).

## 4. Discussion

Early results from several hypofractionated trials [5, 6] indicate that HFRT is safe and provides good biochemical control. We reported our experience of favorable risk localized prostate carcinoma patients treated with hypofractionated radiotherapy schedule 60 Gy/20 fractions over 4 weeks associated with IGRT. This group of patients was compared with a group of 32 low-risk patients treated with conventional fractionation and IGRT that refused hypofractionated treatment.

The actuarial 4-year OS was 100% and 93.8% (*P* = 0.053) for the HFRT and CFRT groups, respectively. Two deaths occurred in the CFRT group at the time of analysis. The patients died after 7 and 10 months from RT completion, respectively, for cardiopulmonary disorders without any evidence of disease. The actuarial 4-year bNED and DSS were 97.4% for HFRT group. The actuarial 4-year bNED and DSS were 100% for the CFRT group. There was no significant difference (*P* = 0.374) between the two groups. One patient developed biochemical failure with evidence of external iliac lymph nodes involvement. Our results were favorable compared to other experiences of dose escalation using conventional fractionation regimens. In the literature, there are two randomized trials that reported long-term outcomes. Zietman et al. [7] had shown 5-year bNED rates of 91% and 10-year bNED rates of 84%. Kuban et al. [8] reported 5- and 8-year bNED rates of 100% and 88%, respectively, in low-risk prostate cancer patients.

A randomized study by Pollack et al. [9] reported no significant difference in terms of bNED between the two arms of patients treated with hypofractionated regimen versus standard fractionation, even though every type of prostate cancer risk was included in the study. Another study by Martin et al. [10] reported similar outcomes to our study: the 3-year bNED was 100% in low-risk patients who underwent IGRT-IMRT hypofractionated radiotherapy. Patel et al. [11] reported actuarial biochemical control rates and cancer-specific survival at 5 years of 97% and 100%, respectively, using 3-dimensional conformal radiotherapy with total dose of 66 Gy delivered in 22 fractions in low-intermediate-risk prostate cancer patients.

Treatment was well tolerated with more than 50% of patients presenting no acute urinary or gastrointestinal toxicity. The incidence of acute toxicity in our cohort was lower than other series. Martin et al. [10] reported 11% of grade 2 GI acute toxicity and 25% of grade 2 GU acute toxicity after HFRT with 60 Gy in 20 fractions in localized prostate cancer patients. Soete et al. [12] reported 5% of grade 2 acute GI toxicity with no grade 3 toxicity using 56 Gy/16 fractions regimen. In our study, toxicities during treatment occurred as follows: grade 1-2 acute GI toxicity in 17.6% patients; grade 1-2 and grade 3 acute GU toxicity in 48.4% and 1.1% patients, respectively. In a recent study by Patel et al. [11] the reported toxicity rates in patients with favourable risk prostate cancer who underwent 66 Gy/22 fractions were 17% of grades 1-2 GI toxicities and 33% of grade 1-2 GU toxicities; only 1% of patients experienced grade 3 GU toxicity. Pollack et al. [13] using 70.2 Gy in 26 fractions (2.7 Gy per fraction) found, at 3 months after RT, 6% grade 2 GU toxicity and no grade 2 GI toxicity. At 3 months after radiation treatment we observed grade 1 GI and GU toxicity in 5.1% and 10.2% of patients treated with HFRT, respectively. No grade ≥2 GU and GI toxicities were recorded. During the treatment grade 2-3 GU toxicity rates were low but resulted significantly higher (*P* = 0.04) in the group treated with CFRT. No statistically significant difference in acute toxicity was calculated between the two groups treated with CFRT and HFRT at 3 months from the end of therapy. As this comparison study is retrospective in nature, it is subject to the biases of this methodology.

One major concern about hypofractionation regimen with a high BED is the manifestation of potential late effects. Toxicity was prospectively scored in every patient at each follow-up visit. Our long-term results demonstrated that HFRT regimen was well tolerated. At 6 months from the end of therapy, 2 patients (3.4%) presented grade 1 GI toxicity and 7 patients (11.9%) presented grade 1 GU toxicity. No grade ≥2 toxicities were recorded.

Kupelian et al. [14] reported grade 1-2 of late GI toxicity in 9%, grade 3-4 in 1.4% of the patients, grade 1-2 of late GU toxicity in 9.4%, and grade 3 in 0.1% after conventional radiotherapy with 70 Gy in 28 fractions of 2.5 Gy with a 4 mm rectal margin. Pollack et al. [13] reported 5.9% of late grade ≥2 GI toxicity after 70.2 Gy in 26 fractions of 2.7 Gy. A recent update of the same trial described higher GU toxicities in the HFRT arm (18.3% versus 8.3%, *P* = 0.028) compared to the CFRT arm [9]. Martin et al. [10] described an incidence of 6.3% of late grade ≥2 GI toxicity and 4.3% of late grade ≥2 GU toxicity after hypofractionated regimen (60 Gy/20 fractions). After 2 and 5 years of follow-up, several studies reported grade ≥2 late GI side effects of 4% and 5.5% and late bladder side effects in 4.2% and 5.6%, respectively [15, 16]. Rene et al. [17] reported at the last follow-up persistent grade ≥2 late GU and GI toxicity of 2% and 1.5%, respectively. In the current study, at the last follow-up grade 1 GI and GU toxicities were observed in 5 patients (8.5%) and 8 patients (13.6%), respectively. No grade ≥2 toxicities were recorded. The actuarial incidence of grade 1-2 GI toxicities at 4 years was 13.6%, for CFRT group was 7.2%, and for HFRT group was 24.5% (*P* = 0.191). The actuarial incidence of grade 1-2 GU toxicities at 4 years was 11.5%, for CFRT group was 4%, and for HFRT group was 49% (*P* < 0.001). Patients treated with HFRT during the follow-up had more probability to develop transient event of grade 1-2 GU toxicity. In fact, most of these symptoms resolved over time and 84% of patients presented no GU symptoms at the last follow-up. The lower toxicity rates achieved in our study, especially GI, are likely a result of the advantage of reduced margins from CTV to PTV combined to daily IGRT. Although today IMRT has been widely adopted as the radiation technique of choice for prostate cancer according to better sparing of the bladder and rectum, our study demonstrated that hypofractionated radiation therapy can be safely delivered using IGRT-3D-CRT. In fact, we reported a toxicity rate similar to another study in which hypofractionated regimens were delivered with IMRT.

Men treated with 60 Gy/20 fractions in our study experienced acceptable toxicity rates. From our data, there appears to be no increase in late toxicity, using a 60 Gy hypofractionated regimen for localized prostate cancer. There are three ongoing phase 3 randomized trials attempting to evaluate the effectiveness and tolerance of hypofractionated RT regimens compared with standard RT fractionation. The Canadian Prostate Fractionated Irradiation Trial (PROFIT) compared the Princess Margaret Hospital regimen of 60 Gy in 20 fractions [10] with standard regimen 78 Gy in 39 fractions in intermediate-risk patients. Up to 6 months of hormonal treatment before radiation therapy was allowed. The RTOG 0415 trial assessed HypoRT in low-risk patients without hormonal therapy. This study tested Kupelian's [14] regime of 70 Gy in 28 fractions versus 73.8 Gy in 41 fractions. Both trials are completed, but neither has reported preliminary results already. The British CHHiP (Conventional or Hypofractionated High Dose Intensity Modulated Radiotherapy for Prostate Cancer) [18] included a range of patients with localized prostate cancer even those with clinical stage T 1–3, PSA up to 30 ng/mL, and any Gleason score. This trial compared the standard fractionated schedule of 74 Gy in 37 fractions with 2 hypofractionated regimes (60 Gy/20 fractions and 57 Gy/19 fractions) in patients with low-, intermediate-, and high-risk disease. Short course hormonal therapy for 3–6 months was administered to the majority of patients. The interim analysis of this study has shown that grade ≥2 GI toxicity at 2 years in the conventional versus 60 Gy group versus 57 Gy group was 4.3%, 3.6%, and 1.4%, respectively, and grade ≥2 GU toxicity was 2.2%, 2.2%, and 0, respectively.

Further, technology has evolved dramatically since the publication of the earliest HFRT studies. Some retrospective studies show that such novel technologies may improve outcomes, but others show no effect in the context of HFRT [19, 20]. Image-guidance and prostate immobilization have become a critical component of stereotactic body radiation therapy (SBRT) because there is potential of large doses delivered to prostate cancer [21, 22]. SBRT and hadron therapy are acquiring acceptance as alternative to CFRT and HFRT schedules. No phase III data are available, although the current literature includes several phase I/II trials.

## 5. Conclusions

Our study demonstrated that hypofractionated regimen provided excellent biochemical control in favorable risk prostate cancer with the 4-year actuarial biochemical control rate of 97.4%. The incidence of acute and late GI and GU toxicity was low. However, in our study HFRT presented higher cumulative incidence of low-grade late GU toxicity than CFRT.

Results from completed randomized phase 3 trials will be necessary to allow us to define the *α*/*β* ratio of prostate cancer more accurately and confirm whether hypofractionation is a safe method of delivering dose-escalated curative radiation therapy.

## Figures and Tables

**Table 1:** Patients' characteristics.

Characteristics	HFRT (*n* = 59)		CFRT (*n* = 32)		Total (*n* = 91)	
	*n*	%	*n*	%	*n*	%
Age						
<70	19	32.2	4	12.5	23	25.3
≥70	40	67.8	28	87.5	68	74.7
PSA at diagnosis (ng/mL)						
0.1–5	19	32.2	9	28.1	28	30.8
5.1–9.9	40	67.8	23	71.9	63	69.2
Gleason score						
6	59	100	32	100	91	100
Clinical stage						
T1c	17	28.8	8	25.0	25	27.6
T2a	42	71.2	24	75.0	66	72.4

**Table 2:** Comparison of acute GI and GU toxicities in the CFRT group versus HFRT group.

Toxicity	GI					GU				
	CFRT		HFRT		*P* value	CFRT		HFRT		*P* value
	*n*	%	*n*	%		*n*	%	*n*	%	
During RT										
G1	7/32	21.9	7/59	11.9	0.206	15/32	46.9	23/59	39.0	0.466
≥G2	2/32	6.3	0/59	0	0.052	6/32	18.8	1/59	1.7	0.04
3-month FU										
G1	2/32	6.3	3/59	5.1	0.816	3/32	9.4	6/59	10.2	0.904
≥G2	0	0	0	0		0	0	0	0	
